# A Comparative Serological Study of Toxoplasmosis in Pregnant Women by CLIA and ELISA Methods in Chalus City Iran

**DOI:** 10.5812/ircmj.15115

**Published:** 2014-04-05

**Authors:** Zahra Elahian Firouz, Hami Kaboosi, Abdolreza Faghih Nasiri, Seyed Saleh Tabatabaie, Farideh Golhasani-Keshtan, Fatemeh Zaboli

**Affiliations:** 1 Department of Microbiology, Islamic Azad University, Science and Research Ayatollah Amoli Branch, Amol, IR; 2 Department of Infectious Disease, Iran Blood Transfer Organization, Sari, IR Iran; 3 ENT-HNS Research Center, Hazrate Rasool Hospital, Iran University of Medical Sciences, Tehran, IR Iran; 4 Department of Physiology, Mashhad University of Medical Sciences, Mashhad, IR Iran; 5 Department of Mycology, Islamic Azad University, Science and Research Ayatollah Amoli Branch, Amol, IR Iran

**Keywords:** *Toxoplasma*, Toxoplasmosis in Pregnancy, Congenital Infection, Enzyme-Linked Immunosorbent Assay

## Abstract

**Background:**

Toxoplasmosis is the most common disease in humans and animals (zoonosis) caused by the protozoan parasite *Toxoplasma gondii*. The disease is usually appeared as asymptomatic in immunocompromised individuals but its most common symptom is lymphadenopathy. Shortly before or during the first trimester of pregnancy, this disease can be transferred to the fetus and cause serious infection in the fetus. In late pregnancy (third trimester), the complications of this infection is very low or unsigned. Due to the absence of non-specific clinical symptoms or slight infection in pregnant women, prenatal diagnosis is often impossible.

**Objectives:**

Since no research compared these two methods, we decided to compare these methods and determine which method works better for diagnosis of toxoplasmosis.

**Patients and Methods:**

In this study, 50 pregnant women who referred to the Chalus Health Center laboratory were included and the blood samples were tested for presence of IgG and IgM antibodies of *Toxoplasma gondii* by both ELISA and Chemiluminescence methods.

**Results:**

Of the 50 samples tested by the ELISA method, 26 samples (52%) were positive for IgG . No samples were positive for IgM. Of the 50 samples tested by the Chemiluminescence method, 28 samples (56%) were positive for IgG. No samples were positive for IgM.

**Conclusions:**

A significant relationship between the age of the youngest child and the infection rate was seen. No significant correlation between age, number of individuals in the household, number of children, location, type of construction, consumption of greens, the way of greens and meat consumption, drug use, history of stillbirth and infection levels was seen.

## 1. Background

Toxoplasmosis is the most common disease in humans and animals (zoonosis) caused by the protozoan parasite *Toxoplasma gondii *(1, 2).. This disease remains undiagnosed in normal individuals (3). In pregnant women, it can be transferred from mother to embryo in the first trimester of pregnancy and diagnosed in the newborn after the birth (3), but infection during the last trimester of pregnancy, brings less damage for the embryo (3).Prevalence of toxoplasmosis reported for a wide range of ages and 500 million individuals have serological defection (1-11). For the first time, this diseases was reported in 1948 in Iran (12). Various studies showed different prevalence rates of toxoplasmosis in Iran. The greatest and least prevalence of toxoplasmosis belongs to Rey city and shiraz, respectively (13, 14).Overall, this infection is common in mild and dry foothills of west of Iran with 33-68 percent prevalence rate (15).There are two methods for toxoplasmosis detection, CLIA and ELSA (15).Quantity of IgG and IgM change during infection, therefore, CLIA and ELSA methods can help to recognize toxoplasmosis, these methods are cheap, quick and highly repeatable.

## 2. Objectives

Since no research compared these two methods, we decided to compare these methods and determine which method works better for diagnosis of toxoplasmosis.

## 3. Patients and Methods

Subjects were women who referred to the laboratory of Health center in Chalus city from 2012 till 2013 and had IgG and IgM in their blood. Pregnancy and place of residence were the only criteria for entry in the study. All the blood samples were tested by the CLIA (chemiluminescence) and ELSA (Enzyme-linked immunoadsorbent assay) methods.

### 3.1. Enzyme-linked Immunoadsorbent Assay

This is a simple and highly sensitive biochemical method that analyzes a large number of samples simultaneously. This immunological method detects antibodies or antigens in a sample.

### 3.2. Chemiluminescence

When energy of an excited electron decreases from high to low, the energy released in the form of light and the reaction is known as luminescence. Types of luminescence include fluorescence, phosphorescence and chemiluminescence. Chemiluminescenceis different from other luminescence phenomena in which chemical or electrochemical reactions (not the photoluminescence phenomenon) cause excitement, but this excitement is similar to fluorescence and during electron’s return to baseline energy, light is emitted (16).

## 4. Results

In this study, fifty patients with mean age of 27.4 were entered whose highest education was upper diploma (8 patients). The number of patients from Urban and the country were 21 and 29, respectively. These results summarized in Table 1. Mann-whitney test done for Analysis of variables prevalent in *Toxoplasma *infection. We compared IgG ELISA and IgG Chemi with variables such as education, number of individuals in a household, parity and number of children. The results demonstrated that there is no significant relationship between IgG ELISA and IgG Chemi and different variables (P > 0.05). All results summarized in Table 2. To Comparing the variables with IgG using two methods of ELISA and Chemiluminescence, it was demonstrated that there is no significant difference between variables and methods, except Age of youngest child that has significant difference with IgG ELISA (P = 0.037).These results summarized in Table 3. Overall, mc nemar test demonstrated that there was no significant difference between the two methods, chemiluminescence and ELISA (KAPPA = 0.841).

**Table 1.** Demographic Characteristics of the Patients ^a^

	Results
**Age**	27.4 ± 5.0
**Education**	
Uneducated	1 (2)
Primary	20 (40)
Diploma	21 (42)
Upper diploma	8 (16)
**Lodging**	
Urban	21 (30.4)
Village	29 (42)

^a^ All data are presented as Mean ± SD and No. (%).

**Table 2.** Variables in Prevalence of Infection With *Toxoplasma *^a^

Variables	Positive	Negative	P Value
**Education**			
IgG ELISA	26 (52)	24 (48)	0.36
IgG Chemi	22 (44)	24 (66)	0.33
**Number of individuals in a household**			
IgG ELISA	48 (96)	28 (4)	0.51
IgG Chemi	22 (44)	24 (66)	0.39
**Parity**			
IgG ELISA	48 (96)	28 (4)	0.36
IgG Chemi	22 (44)	24 (66)	0.32
**Number of Children**			
IgG ELISA	48 (96)	28 (4)	0.28
IgG Chemi	22 (44)	24 (66)	0.32

^a^ Data are presented as No. (%).

**Table 3.** To Compare Variables with IgG Using twoMethods ofELISA and Chemiluminescence ^a^

Variable	Positive	Negative	P Value
**IgG ELISA**			
Age	28.77 ± 4.893	26 ± 4.908	0.052
Age of abortion	29.67 ± 3.055	27 ± 6.557	0.558
Age of youngest child	5.31 ± 3.172	8.33 ± 3.041	0.037
**IgG chemiluminescence**			
Age	28.36 ± 4.249	26.71 ± 5.563	0.256
Age of abortion	29.67 ± 3.055	27 ± 6.557	0.558
Age of youngest child	5.64 ± 3.295	7.45 ± 3.417	0.218

^a^ Data are presented as Mean ± SD.

## 5. Discussion

According to our results from the ELIZA method, the frequency of IgG in patients was 26 out of 50 individuals, and there was no frequency of IgM. Therefore, the results of chemiluminescence method are the same as the results from ELIZA. These results demonstrate that toxoplasmosis had a high frequency in the studied area. In this study, infection was diagnosed in more than half of the subjects; but fortunately, none of them showed acute infection. If there are not factors weakening the immune system and made Igm negative, so there is no risk for disease transmission from mother to the fetus.

In a study by Bouer et al. two methods of ELISA and IFA (The Indirect Fluorescence Assay) were used for detection of IgM and IgG antibodies of *Toxoplasma*. The results demonstrated that antibody which used ELISA and IFA methods in infected cases was detected after three and nine days, respectively. Also, the frequency of IgM and IgG was different in these two methods. Detection of antibodies using the ELISA test is more sensitive than ones using the IFA test (17), therefore we decided to use the ELISA test in this study. In the study performed by Veeranootet al, ELISA test was used, and it was demonstrated that IgG was positive and IgM was negative in pregnant women (18); this is similar to the findings of our study. In another study, Zamora et al. used the chemiluminescent immunoassay (CLIA) for detection of *Salmonella* antibodies and compared this with the ELISA method. In this study, LPS antigen detection was tested. Comparing the method of ELISA and CLIA, it was demonstrated that detection via the ELISA method is more careful than the CLIA method, because lipopolysaccharide antigen reaction with *Escherichia coli* and Yersinia strains was found when it was tested with ELISA but this was not observed using the CLIA test. Therefore, due to the wide detection range of CLIA, it can be used for detection of *Salmonella* antibodies (19).However, there are many studies in which CLIA was used for detection of *toxoplasma*. In the study of Nissapatorn et al. comparing the methods of ELISA and PCR, it was revealed that IgG in toxoplasmic patients was positive using the ELISA method but not PCR (18);this result indicates that ELISA is more sensitive that PCR, and ELSIA is better than PCR for detection of toxoplasmosis. In the other study done by Ajami et al., it was demonstrated that the prevalence of toxoplasmosis depends on the age, and increasing the age can increase the prevalence of toxoplasmosis (20); this study was done on women before marriage, but our study was done on pregnant women and the results demonstrated that there is no relationship between age and toxoplasmosis using the two methods. In the study conducted by Ebrahimzadeh et al, there was a significant relationship between age and education, but our results showed the opposite. Age of pregnant women is limited therefore there is probably no significant difference in age of participations which could affect the prevalence of toxoplasmosis. Overall, as other studies, the present study also showed that the CLIA method is more sensitive than ELISA, because percentage of IgG in CLIA and ELISA was 58% and 52%, respectively. Since CLIA was automatically done by a machine and not by human, therefore, error could be decreased. Therefore, CLIA is the best method for detection of toxoplasmosis and antibodies in toxoplasmosis.
